# Necrostatin-1 Prevents Necroptosis in Brains after Ischemic Stroke via Inhibition of RIPK1-Mediated RIPK3/MLKL Signaling

**DOI:** 10.14336/AD.2018.0728

**Published:** 2019-08-01

**Authors:** Xu-Xu Deng, Shan-Shan Li, Feng-Yan Sun

**Affiliations:** ^1^Department of Neurobiology, School of Basic Medical Sciences and National Clinical Research Center for Aging and Medicine, Huashan Hospital, Shanghai Medical College, Fudan University, Shanghai, China; ^2^Institute for Basic Research on Aging and Medicine, the State Key Laboratory of Medical Neurobiology, School of Basic Medical Sciences, Fudan University, Shanghai, China; ^3^Shanghai Key Laboratory of Clinical Geriatric Medicine, Huadong Hospital, Fudan University, Shanghai, China

**Keywords:** RIPK1, necroptosis, necrostatin-1 (Nec-1), cerebral stroke, inflammation, neuroprotection

## Abstract

Pharmacological studies have indirectly shown that necroptosis participates in ischemic neuronal death. However, its mechanism has yet to be elucidated in the ischemic brain. TNFα-triggered RIPK1 kinase activation could initiate RIPK3/MLKL-mediated necroptosis under inhibition of caspase-8. In the present study, we performed middle cerebral artery occlusion (MCAO) to induce cerebral ischemia in rats and used immunoblotting and immunostaining combined with pharmacological analysis to study the mechanism of necroptosis in ischemic brains. In the ipsilateral hemisphere, we found that ischemia induced the increase of (i) RIPK1 phosphorylation at the Ser166 residue (p-RIPK1), representing active RIPK1 kinase and (ii) the number of cells that were double stained with P-RIPK1 (Ser166) (p-RIPK1^+^) and TUNEL, a label of DNA double-strand breaks, indicating cell death. Furthermore, ischemia induced activation of downstream signaling factors of RIPK1, RIPK3 and MLKL, as well as the formation of mature interleukin-1β (IL-1β). Treatment with necrostatin-1 (Nec-1), an inhibitor of necroptosis, significantly decreased ischemia-induced increase of p-RIPK1 expression and p-RIPK1^+^ neurons, which showed protection from brain damage. Meanwhile, Nec-1 reduced RIPK3, MLKL and p-MLKL expression levels and mature IL-1β formation in Nec-1 treated ischemic brains. Our results clearly demonstrated that phosphorylation of RIPK1 at the Ser166 residue was involved in the pathogenesis of necroptosis in the brains after ischemic injury. Nec-1 treatment protected brains against ischemic necroptosis by reducing the activation of RIPK1 and inhibiting its downstream signaling pathways. These results provide direct *in vivo* evidence that phosphorylated RIPK1 (Ser 166) plays an important role in the initiation of RIPK3/MLKL-dependent necroptosis in the pathogenesis of ischemic stroke in the rodent brain.

Stroke, an aging related disease, is the first leading cause of disability and major cause of death in the world. The pathogenesis of neuronal damage in the brain after ischemic stroke is highly complex, involving necrotic, apoptotic and autophagic processes [1, 2]. The major distinction between apoptosis and necrosis is that apoptosis of cells follows a programmed pathway that is positively regulated by caspase-dependent signaling factors, whereas necrosis is not programmed and passively triggered by excitotoxity [3]. However, recent evidence have clearly demonstrated that necrotic cell death can also be regulated by the death domain receptor-associated adaptor kinase while caspase-8 is inhibited, which is referred as to necroptosis or programmed necrosis [4, 5]. Pharmacological analysis has revealed that treatment with necrostatin-1 (Nec-1), a necroptosis inhibitor [5, 6], could reduce neuronal death and infarct volume in animal brains using different models, such as middle cerebral artery occlusion (MCAO) [5, 7], traumatic brain injury (TBI) [8], controlled cortical impact (CCI) [9] and hypoxia-ischemia (HI) [10-12]. These observations indirectly suggested that necroptosis might be involved in the pathogenesis of neuronal death after various injuries to the brain. Presently, there is no study showing biochemical and morphological evidence that directly demonstrates whether signaling factors of the necroptotic pathway are indeed involved in such pathological processes.

Necroptosis is a caspase-independent regulated type of cell death that relies on the formation of a necrosome, which is mainly composed of the receptor-interacting protein kinase-1 (RIPK1), receptor-interacting protein kinase-3 (RIPK3) and mixed lineage kinase domain-like protein (MLKL). Activation of RIPK1 kinase is one of the initiators of the necroptosis pathway. RIPK1, a 74 kDa protein, is composed of a N-terminal kinase domain, an intermediate domain (containing the RIP homotypic interaction motif, RHIM) and a C-terminal death domain [13, 14]. When tumor necrosis factor-α (TNFα) binds the TNF receptor-1 (TNFR-1) and forms the associated signaling complex (Complex I) on the membrane, RIPK1 is recruited and then phosphorylated at the appropriate serine residues. Activation of RIPK1 through its phosphorylation in turn activates RIPK3 and MLKL kinases to form Complex IIb under condition of caspase-8 inhibition, which results in necroptosis [15, 16].

It has been reported that kinase activation of RIPK1 manifests as auto-phosphorylation [6]. In *in vitro* assays, Nec-1 protects cells from necroptotic death via a reduction of RIPK1 kinase activity through inhibition of RIPK1 serine-phosphorylation at several residues [17]. For example, in HEK293T cells, Yuan and colleagues demonstrated that TNFα induces cell necroptosis and the phosphorylation of RIPK1 at the Ser166 residue i.e. p-RIPK1 (Ser166); both of which can be effectively inhibited by Nec-1 [18]. Therefore, p-RIPK1 (Ser166) is considered a biomarker for the activation of RIPK1 kinase and necroptosis [18, 19].

In this study, we used a MCAO model in rats combined with different approaches to investigate the possible roles of RIPK1-triggered RIPK3/MLKL-dependent necroptosis in the pathogenesis of ischemic brain injury. We found that cerebral ischemia stimulated phosphorylation of RIPK1 at the Ser166 residue and increased RIPK3 and MLKL expression levels in the brain. Moreover, Nec-1 could effectively inhibit ischemia-induced changes described above while it protected the brain against ischemic injury and functional deficits. Our data suggest that (i) p-RIPK1 (Ser166) participated in RIPK3/MLKL-dependent neuronal necroptosis after cerebral ischemia; (ii) immunostaining with a specific antibody against p-RIPK1 (Ser166) could be used as a morphological biomarker for necroptosis. This study also brings new insights into the pathological mechanism of neuronal damage in the brain after ischemic stroke.

## MATERIALS AND METHODS

### Animals

Adult male Sprague-Dawley rats (220-250g) were purchased from the Sino-British SIPPR/BK Laboratory Animal Ltd (Shanghai, China) All experimental procedures followed the National Institutes of Health Guide for the Care and Use of Laboratory Animals Approval of the study protocol was granted by the Ethics Committees of Experimental Research of the Shanghai Medical College of Fudan University (Permit Number: 20120302-128) All surgeries were performed under anesthesia and all efforts were made to minimize suffering and reduce the number of animals used.

### Transient focal cerebral ischemia model

Unilateral transient focal cerebral ischemia was induced by MCAO as described previously [20]. Animals were anesthetized with 10% chloral hydrate (360 mg/kg, i.p.). Only animals within the normal range of arterial blood pO2, pCO2 and pH, measured by an i-STAT Blood Gas Analyzer (Abbott Laboratories, Chicago, USA), were included in the study. Rectal temperature was maintained at 37 ± 0.5 ºC during surgery with a heating pad. In brief, a 4-0 nylon monofilament with a rounded tip was introduced into the left external carotid artery stump and gently advanced into the internal carotid artery until a slight resistance was felt. The filament was left in place for 30 min and then withdrawn to allow for reperfusion. The sham-operated animals were treated identically, except there was no occlusion of the middle cerebral artery after the neck incision. Regional cerebral blood flow was monitored by a Laser Doppler perfusion monitor (Periflux system 5000, Terimed AB, Sweden) during pre-ischemia, ischemia, and reperfusion to verify the success of the cerebral ischemia/reperfusion procedure. A decrease in blood flow to 20% of baseline indicates successful MCA occlusion.

### Experimental groups and administration of necrostatin-1

All animals were randomly divided into three groups: a sham-operated group (Sham); an inactive necrostatin-1 treated control group (iNec); and a necrostatin-1 treated group (Nec-1). Necrostatin-1 and inactive necrostatin-1 purchased from Chemicon (CA, USA) were stereotaxically delivered into the contralateral ventricle (AP, 0.8 mm; ML, 1.4 mm; DV, 3.6 mm) at a volume of 1.5 μl (20 mM, dissolved in 10% DMSO) 30 min before MCAO.

### Evaluation of neurological function

Neurological assessments were conducted at four time-points: 1 h before MCAO (pre-) and 12, 24, 72 h after the onset of ischemia.

Neurological deficits were scored on a five-point scale according to Longa et al. [21]: 0, no neurological deficits; 1, failure to extend the contralateral forelimb fully when lifting the animal by the tail; 2, contralateral circling; 3, leaning to contralateral side at rest; 4, no spontaneous motor activity.

For the forelimb placing test [22], rats were held by their torsos to make forelimbs hang freely and then brushed against the desktop edge. Normal rats would quickly place the forelimb of both sides on the desktop, while rats with brain injury could not place the contralateral forelimb on the desktop edge successfully. The rats were tested 10 times and scores were represented by the number of times the forelimbs were placed on the edge of the table.

### Brain section preparation

Animals were deeply anesthetized with 10% chloral hydrate (360 mg/kg, i.p.) and quickly transcardially perfused with saline followed by 4% paraformaldehyde (PFA) dissolved in 0.1 M phosphate-buffer (pH 7.4). Brains were removed and post-fixed in 4% PFA for 12 h and successively equilibrated in 20% and 30% phosphate-buffered sucrose solutions. The brains were then embedded using Tissue-Tek (OCT compound, Sakura, CA, USA) and frozen at -20 ºC. Serial coronal sections were obtained (30 μm thick, Bregma 1.0 mm to -0.2 mm) using a freezing microtome (Model 820-II, Leica, Wetzlar, Germany) and stored at -20 ºC in cryoprotectant solution.

### Assessment of infarct volume

Serial coronal sections for Cresyl violet (Sigma, MO, USA) staining were collected (30 μm thick at 360 μm intervals, 1.60 to -4.80 mm from Bregma) when preparing brain sections as described above. The infarct areas were recorded with a digital camera using a 1.25× objective and analyzed with Adobe Photoshop CS6 (V13.0, Adobe Systems, CA, USA). The percentage of the infarct volume was calculated by the following formula: [(total contralateral hemispheric volume)-(total ipsilateral hemispheric stained volume)]/(total contralateral hemispheric volume) × 100%.

### Immunohistochemistry and immunofluorescence

Brain sections were incubated with rabbit monoclonal anti-phospho-RIPK1 (Ser166) (1:100, 65746S, Cell Signaling, MA, USA) antibody overnight at 4 ºC followed by incubation with corresponding biotinylated secondary antibody (Vector Laboratories, CA, USA) and avidin-biotin-peroxidase complex (Vector Laboratories). Immunoreactivity was detected with 0.05% diaminobenzidine (Sigma) solution. For double staining, sections were incubated with mouse monoclonal anti-NeuN (1:200, ab13938, Abcam, Cambridge, UK) and immunoreactivity was detected with the Vector® Blue AP Substrate Kit (Vector Laboratories). Incubation without the primary antibody served as negative control where no immunoreactive signal was detected.

For immunofluorescence, brain sections were incubated with anti-phospho-RIPK1 (Ser166) antibody overnight at 4 ºC followed by incubation with anti-rabbit IgG-594 (1:1000, A21207, Life Technologies, MA, USA) antibody for 1 h at 37 ºC. Nuclei were counterstained with DAPI at room temperature for 15 min. The fluorescent signals were detected at excitation 535 nm and emission 565 nm (594), 380 nm and 530 nm (DAPI), using confocal laser scanning microscopy (TCS SP5, Leica, Wetzlar, Germany).

Sections underwent the same fixation, sectioning, and immunostaining procedures described above in preparation for cell counting. NeuN-p-RIPK1 double-labeled cell counting was performed using a light microscope (DMI6000B, Leica, Wetzlar, Germany) with a 20× objective, digitizing four views randomly chosen from the peri-ischemic area in the ipsilateral and contralateral striatum.

### TUNEL staining

TUNEL staining was performed to obtain fluorescein-labeling according to the manufacturer’s instructions in the In Situ Cell Death Detection Kit (Roche Diagnostics, Mannheim, Germany). Immunofluorescence was then performed using the anti-phospho-RIPK1 (Ser166) antibody according to the procedure above to obtain a double stain with TUNEL-positive cells. TUNEL-positive cells were observed at excitation 488 nm and emission 525 nm using confocal laser scanning microscopy (TCS SP5, Leica).

### Western blotting

Snap-frozen brain tissue was homogenized with ice-cold RIPA lysis buffer and the supernatant was collected as previously described [20]. 20 μg of total protein lysate was used for SDS-PAGE and transferred onto PVDF membranes (Bio-Rad, CA, USA). The membranes were incubated with mouse monoclonal anti-RIPK1 (1:1000, 610458, BD Pharmingen, CA, USA), rabbit monoclonal anti-phospho-RIPK1 (Ser166) (1:1000, 65746S, Cell Signaling), rabbit polyclonal anti-RIPK3 (1:1000, ab56164, Abcam), rat monoclonal anti-MLKL (1:1000, MABC604, Merck Millipore, MA, USA), rabbit monoclonal anti-phospho-MLKL (Ser345) (1:1000, 37333S, Cell Signaling), mouse monoclonal anti-IL-1β (1:1000, MCA1397, AbD Serotec, Kidlington, UK), rabbit polyclonal anti-H3 (1:2000, 9715S, Cell Signaling) or mouse monoclonal anti-GAPDH (1:10000, KC-5G4, KANGCHEN, Shanghai, China) antibody overnight at 4 ºC, and then incubated with anti-mouse/rabbit IgG peroxidase-conjugated secondary antibody (Santa Cruz, CA, USA). Signal was visualized with Western blotting Luminol Reagent (Santa Cruz), detected using ChemiDoc™ Touch Imaging System (Bio-Rad) and quantified with Image J software (V1.48, National Institutes of Health, USA). Beta-actin (1:10000, A5441, Sigma) was used for signal normalization.

### Dephosphorylation

A total of 20 μg protein lysate was collected as above and mixed with Calf Intestinal Alkaline Phosphatase (CIP) (2 μl/200 μl total reaction volume, NEB, MA, USA). For the dephosphorylation reaction, we incubated the mixture at 37 ºC for 30 min followed by precipitation with precooled acetone.

**Figure 1.**
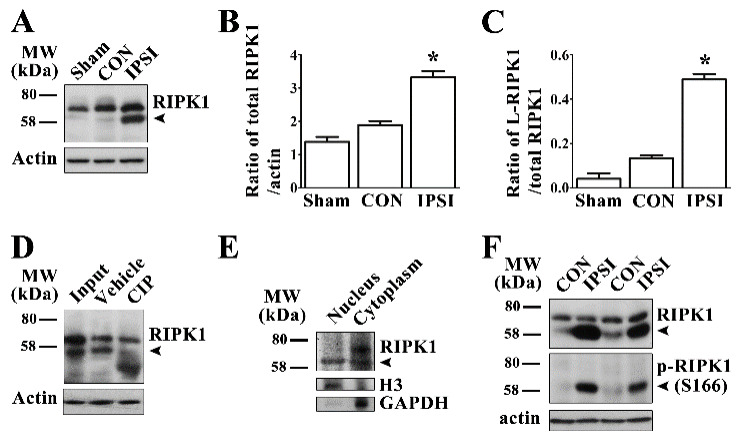
Expression and identification of phosphorylated RIPK1 in rat brains after ischemic injury (**A**) Expression levels of RIPK1 in sham-operated (Sham) and ipsilateral (IPSI)/contralateral (CON) striatum at 24 h after MCAO. Expression levels of total RIPK1 (B) and low molecular weight RIPK1 (C) increased in the ipsilateral striatum compared with the contralateral striatum and sham-operated rats (n≥6). *p<0.05. (**D**) De-phosphorylation of RIPK1 in the ipsilateral striatum at 24 h after MCAO by calf intestinal alkaline phosphatase (CIP) treatment. (**E**) Expression levels of RIPK1 in the nuclear and cytoplasmic compartments of cells in the ipsilateral striatum, H3 was used as a specific nuclear marker and GAPDH as a specific cytoplasmic marker. (**F**) L-RIPK1 immuno-reacted with p-RIPK1 (Ser166) monoclonal antibody.

**Figure 2.**
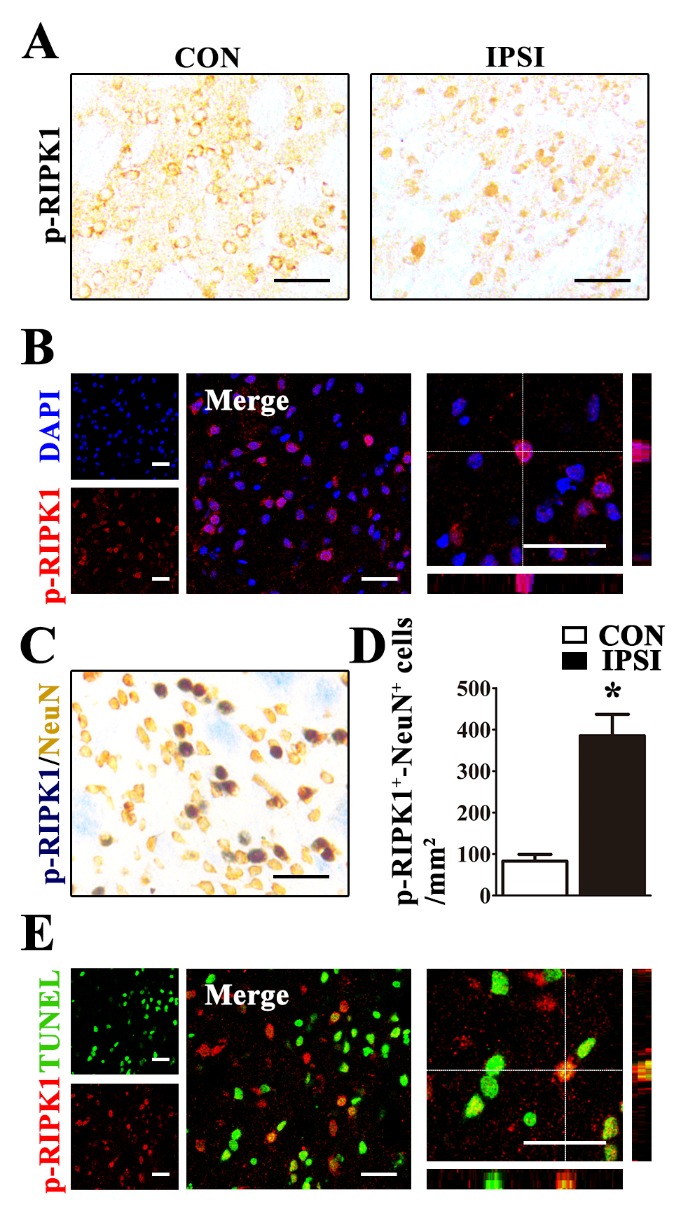
Distribution of phosphorylated RIPK1 (Ser166) in the ischemic rat brain (**A**) Morphology of p-RIPK1 positive (p-RIPK1^+^) cells in the contralateral (CON) and the ipsilateral (IPSI) striatum at 24 h after MCAO. (**B**) p-RIPK1^+^ (red) signals co-localized with DAPI (blue) in the ipsilateral striatum of rats at 24 h after MCAO. (**C**) p-RIPK1 and NeuN double positive (p-RIPK1^+^-NeuN^+^) neurons in the ipsilateral striatum at 24 h after MCAO. (**D**) The number of p-RIPK1^+^-NeuN^+^ neurons increased in the ipsilateral (IPSI) compared with the contralateral (CON) striatum at 24 h after MCAO (n=5). *p<0.05. (**E**) p-RIPK1^+^ signals co-localized with TUNEL stain (green) in the ipsilateral striatum at 24 h after MCAO. Both black and white scale bars in all figures represent 50 μm.

### Nuclear fractionation

The brain tissue was fractionated using the Nuclear and Cytoplasmic Protein Extraction Kit (Beyotime, Jiangsu, China) according to the manufacturer's instructions. Briefly, fresh brain tissue was collected and homogenized with ice-cold cytoplasmic extraction buffer (200 μl/60 mg tissue) then centrifuged (1500 g, 5 min, 4 ºC); the resultant supernatant was the cytoplasm fraction. The precipitate was then resuspended with nuclear extraction buffer, centrifuged (15000 g, 5 min, 4 ºC) and the resultant supernatant was the nuclear fraction.

### Statistical analyses

Experimenters performing drug administration, neurological evaluation and cell counting were blinded to the test conditions until after the study was over.

All experiments were performed at least in triplicates and data are reported as mean ± SEM. Data of neurological scores were analyzed by ANOVA using the PROC GLM of the SAS software (V9.4, SAS Institute, NC, USA). All other data were analyzed using unpaired *t* tests for comparisons between two groups using the GraphPad Prism software (v5.01, GraphPad Software, CA, USA). The criterion for statistical significance was p<0.05.

## RESULTS

### Ischemic injury induced RIPK1 phosphorylation in the ipsilateral striatum of rat brains after middle cerebral artery occlusion

We carried out Western blotting to detect changes of RIPK1 expression level in ischemic rat brains at 24 h after MCAO. The expression level of RIPK1 significantly increased in the ipsilateral striatum (IPSI) compared with that in the sham-operated (Sham) and the contralateral (CON) striatum (Fig. 1A and B). Interestingly, in the ipsilateral striatum, we simultaneously detected a clear low molecular weight band around 58 kDa (L-RIPK1), which was not detected in the contralateral and sham controls (Fig. 1A and C). To determine whether RIPK1 was phosphorylated in the ischemic injured tissues, we further treated samples with calf intestinal alkaline phosphatase (CIP) for protein dephosphorylation before running gel electrophoresis. We observed the disappearance of those L-RIPK1 bands following treatment with CIP, indicating that L-RIPK1 was phosphorylated after cerebral ischemic injury (Fig. 1D). Thereafter, we performed an immunoblotting assay with nuclear and cytoplasmic extraction from ischemic brain tissues and found that L-RIPK1 were mainly detected in the nuclear fraction (Fig. 1E). Furthermore, we used a monoclonal antibody against phosphorylated RIPK1 at the Ser166 residue i.e. p-RIPK1 (Ser166) and found that L-RIPK1 bands, but not original RIPK1 bands, were immunoreacted with the antibody against p-RIPK1 (Ser166) (p-RIPK1, Fig. 1F). Taken together, these results suggest that cerebral ischemic injury induced RIPK1 phosphorylation at the Ser166 residue.

**Figure 3.**
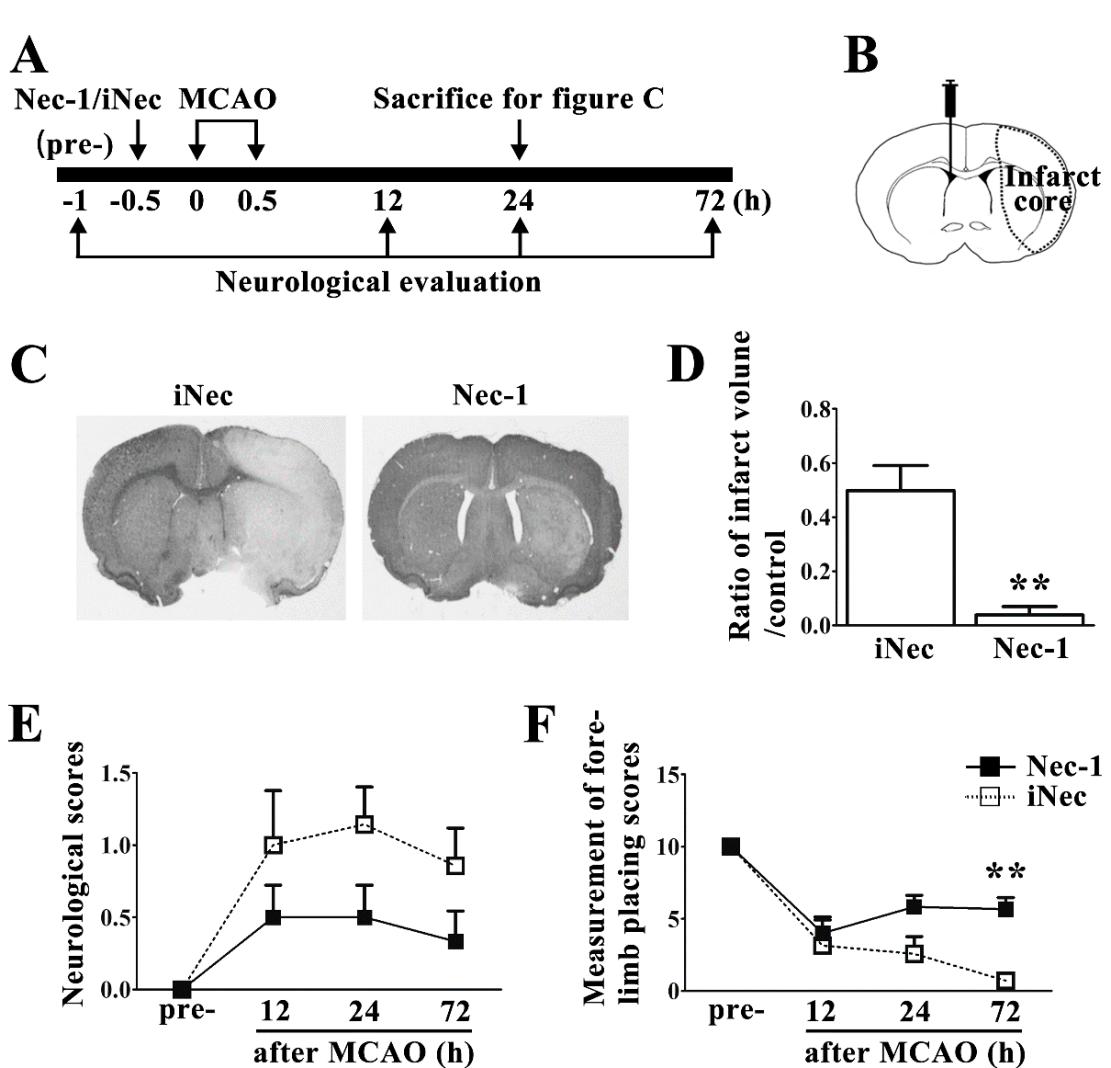
Necrostatin-1 treatment protected brains against ischemic injury (**A**) Experimental protocol of necrostatin-1 (Nec-1) treatment in MCAO rats. Nec-1/inactive necrostatin-1 (iNec) were stereotaxically injected to the right lateral ventricle 30 min before MCAO surgery. Rat brains were subjected to reperfusion at 30 min after occlusion of the MCA. Neurological evaluations were performed at 1 h before MCAO (pre-) and 12, 24, 72 h after MCAO. (**B**) Schematic of the brain shows the injection position of Nec-1/iNec (contralateral ventricle) and the areas of the infarct core (dotted line) after ischemic injury. (C-D) Nec-1 reduced infarct volume at 24 h after MCAO compared with iNec group (n=6-7). (E-F) Nec-1 reduced neurological impairments after cerebral ischemia. The graphs show neurological scores (E) and forelimb placing scores (F) of rats treated with Nec-1 (black bar) or iNec (white bar). **p<0.01.

### Ischemic injury increased phosphorylation of RIPK1 at the S166 residue in the neurons of rat brains after middle cerebral artery occlusion

We next investigated the distribution of p-RIPK1 (Ser166) by means of immunohistochemical staining of brain sections at 24 h after MCAO. We found that p-RIPK1 (Ser166) positively stained (p-RIPK1^+^) cells exist in both the normal and ischemic injured brain, but the morphology of p-RIPK1^+^ cells were different. In the contralateral hemisphere, p-RIPK1^+^ cells presented a typical morphology being hollow, circular and with short processes, whereas, p-RIPK1^+^ cells in the ipsilateral hemisphere showed an obvious solid circle (Fig. 2A). We further confirmed that this p-RIPK1^+^ signal was mainly in the cell nucleus as indicated by colocalization of p-RIPK1^+^ with DAPI in the cells (Fig. 2B). We also found p-RIPK1^+^ signals in the neurons as indicated by double staining of p-RIPK1 and NeuN (p-RIPK1^+^-NeuN^+^). The number of p-RIPK1^+^-NeuN^+^ neurons were significantly increased in the ipsilateral striatum compared with that of the contralateral (Fig. 2C and D). Therefore, we further performed double immunostaining of p-RIPK1 and TUNEL, a marker for double-strand breaks of DNA, at 24 h after MCAO to study the relationship between p-RIPK1 and DNA damage (Fig. 2E). The results showed that 37.41 ± 4.17% of TUNEL^+^ cells showed p-RIPK1^+^ (p-RIPK1^+^-TUNEL^+^) and 74.29 ± 5.38% of p-RIPK1^+^ cells possessed TUNEL^+^, suggesting that most cells with p-RIPK1 had DNA double-strand breaks.

**Figure 4.**
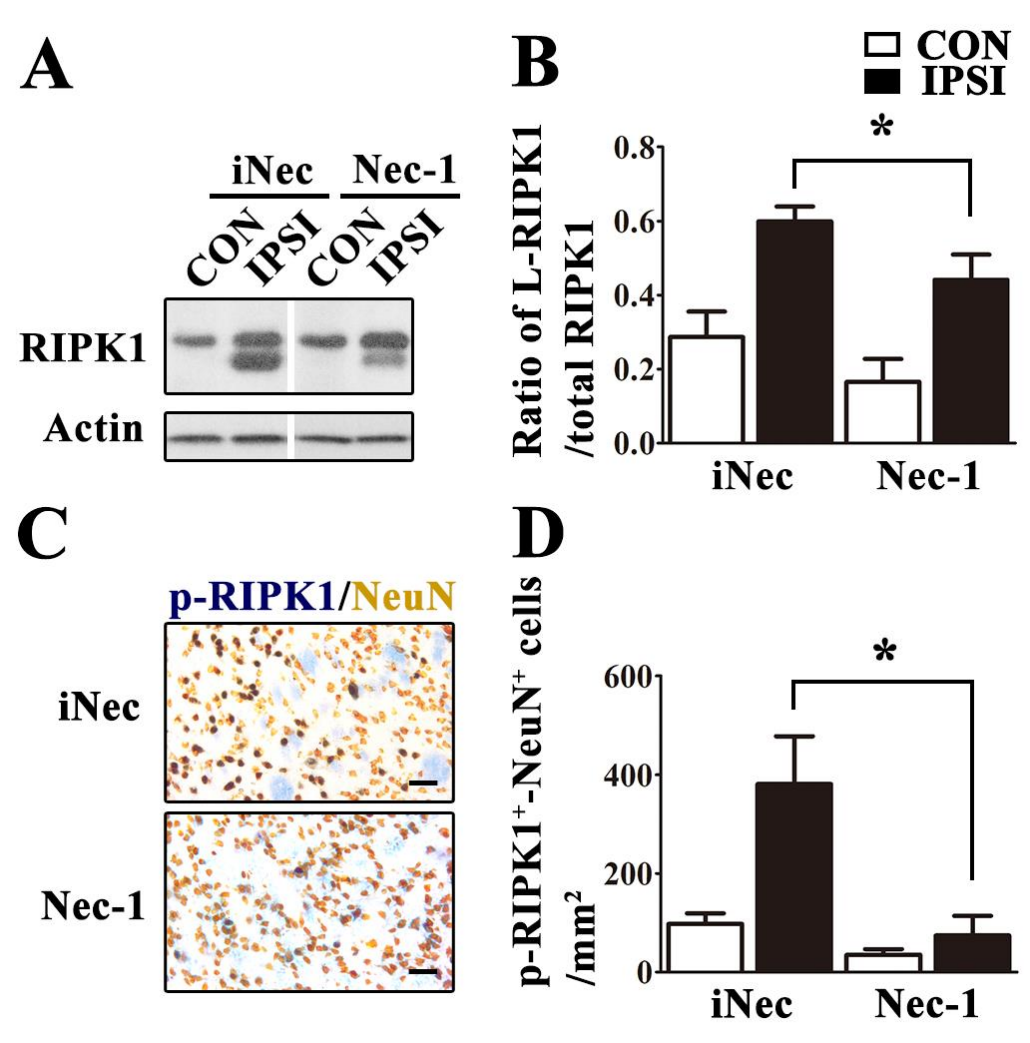
Necrostatin-1 treatment prevented phosphorylation of RIPK1 at Ser166 in neurons after cerebral ischemia (**A-B**) Nec-1 treatment significantly inhibited ischemia-induced increase of p-RIPK1 expression in the ipsilateral striatum (IPSI) at 24 h after MCAO (compared with iNec treatment, p<0.05, n≥10). (**C-D**) Nec-1 reduced the number of p-RIPK1-NeuN double positive (p-RIPK1^+^-NeuN^+^) neurons in the ipsilateral striatum (IPSI) at 24 h after MCAO (compared with iNec treatment, p<0.05, n=5). CON=contralateral striatum. Scale bars indicate 50 μm.

### Necrostatin-1 treatment protected neurons against cerebral ischemic injury after middle cerebral artery occlusion

Previous studies reported that necrostatin-1, an inhibitor of necroptosis, protected neurons against ischemic injury [5, 7]. However, there is still no direct evidence showing whether Nec-1’s protective effect was related to inhibition of RIPK1 kinase activity in cerebral ischemic brains. Therefore, we performed a contralateral intracerebroventricular injection of Nec-1 or inactive necrostatin-1 (iNec) in rats at 30 min before MCAO (Fig. 3A and B). We determined whether Nec-1 injection effectively protected neurons against ischemia-induced death by measuring infarct volume and neurological function in ischemic stroke rats treated with Nec-1 or iNec. We observed that Nec-1 treatment significantly reduced infarct volume (5.08±0.02% in Nec-1 group vs. 45.44±0.1% in iNec group) at 24 h after MCAO (Fig. 3C and D). Neurological deficient scores and forelimb placing scores were evaluated at 1 h before MCAO (pre-) and 12, 24, 72 h after MCAO. The results showed that Nec-1 treatment caused a trend towards the reduction of the neurological deficient scores (Fig. 3E) and a significant enhancement of the forelimb placing scores (Fig. 3F), suggesting that Nec-1 treatment improved the recovery of neurological function in rats after ischemic stroke.

### Necrostatin-1 treatment prevented RIPK1 phosphorylation and its downstream signaling activation

**Figure 5.**
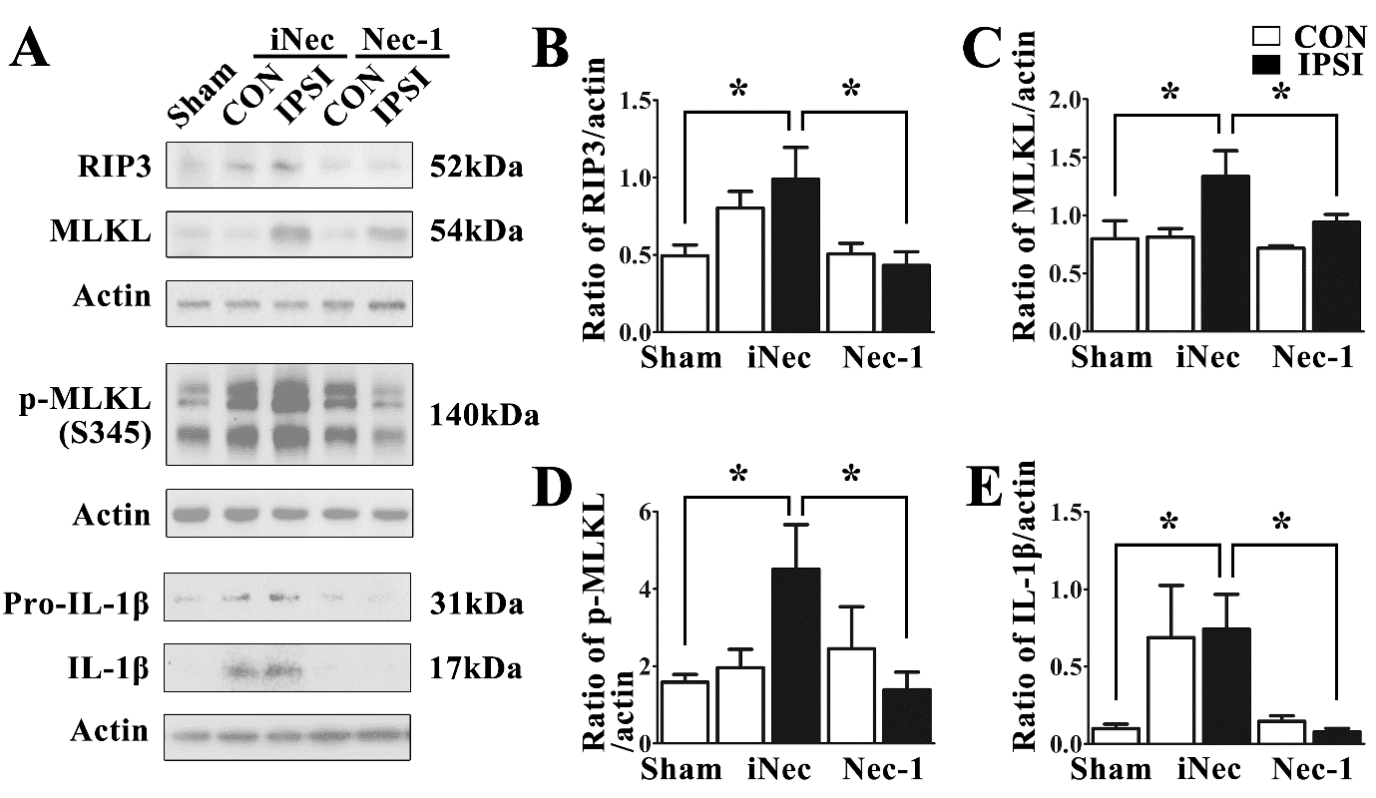
Necrostatin-1 treatment inhibited ischemia-induced expression of RIPK3/MLKL and formation of IL-1β in rat brains after MCAO Expression levels of RIPK3, MLKL, p-MLKL and IL-1β (**A**) in the ipsilateral (IPSI) and contralateral (CON) striatum at 24 h after MCAO. Statistical analysis showed that Nec-1 treatment significantly suppressed RIPK3 (B), MLKL (C), p-MLKL (D) and mature IL-1β (**E**) expression levels in the ipsilateral striatum of MCAO rats compared with iNec-1 treatment (CON), n=4, *p<0.05.

We found that Nec-1 treatment could effectively reduce the amount of L-RIPK1 expression in the ischemic injured brains (Fig. 4A and B) and significantly decrease the number of p-RIPK1^+^-NeuN^+^ double labeled neurons in the ipsilateral striatum (Fig. 4D). These results clearly indicate that stroke-induced RIPK1 phosphorylation plays a critical role in neuronal necroptosis after ischemic stroke. Therefore, we explored the changes of downstream signaling factors of RIPK1-mediated necroptosis in ischemic brains with and without Nec-1 treatment. We used immunoblotting to determine the expression levels of RIPK3 and MLKL, which were the components of the necrosome and that their activities were stimulated by phosphorylated RIPK1 [16, 23]. It was reported that MLKL phosphorylated and formed oligomers to regulate plasma membrane permeabilization, ultimately leading to necroptosis [24, 25]. Therefore, we also detected phosphorylation of MLKL by p-MLKL (Ser345) monoclonal antibody. The results showed that ischemic stroke induced the increase of RIPK3, MLKL and p-MLKL (Ser 345) expression levels in the ipsilateral striatum at 24 h after MCAO (Fig. 5A). Treatment with Nec-1 was observed to simultaneously prevent ischemia-induced increase of RIPK3, MLKL and p-MLKL (Ser345) levels, through its inhibition of RIPK1 kinase activity in the ipsilateral striatum (Fig. 5A, B-D). Furthermore, we also found that ischemic injury induced the formation of mature interleukin-1β (IL-1β), and Nec-1 treatment could significantly suppress mature IL-1β generation (Fig. 5A, E).

## DISCUSSION

In this study, we elucidated the mechanism of necroptosis in the brain after transient cerebral ischemic injury. Necrostatin-1 treatment protected brains against ischemic injury via reducing RIPK1 kinase activity by inhibition of RIPK1 phosphorylation at the Ser166 residue and its downstream signaling factors. We also provide evidence that p-RIPK1 (Ser166) can be used as a biomarker for necroptosis in ischemic/traumatic brain injury.

Activation of RIPK1 kinase induced by TNFα is capable of initiating two cell death pathways (i) RIPK3/MLKL-dependent necroptosis and (ii) FADD (FAS-associated via death domain)/caspase-8-dependent apoptosis [26, 27]. It has been reported that phosphorylation of RIPK1 at the Ser166 residue can be an indicator for activated RIPK1 kinase [18, 19]. Under non-ischemic conditions, RIPK1 kinase is generally inactive [6, 28]. This was supported by our results wherein activation of RIPK1 kinase was almost undetectable in the contralateral hemisphere as well as sham-operated animals (Fig. 1A and C). Interestingly, we found that transient cerebral ischemia could significantly activate RIPK1 kinase as indicated by increase of p-RIPK1 (Ser 166) in injured brain tissues (Fig. 1F). Based on the results from immunoblotting combined with the CIP assay, we found that L-RIPK1 contained p-RIPK1 (Ser166) (Fig. 1A, D and F). Results from the Nec-1 treatment further supported this finding (Fig. 4A). Furthermore, we found that p-RIPK1 (Ser166) was mainly localized to the cell nucleus in ischemic brains (Fig. 1E and Fig. 2A-B), suggesting that p-RIPK1 translocated to the nucleus in times of cellular stress. Taken together, the increase in p-RIPK1 (Ser166) expression level and its cellular location are associated with RIPK1-mediated necroptosis in ischemic brain injury.

We also demonstrated that ischemia-induced phosphorylation of RIPK1 is a specific indicator for necroptosis. We found that in the cells with p-RIPK1^+^, more than 70% of them were also TUNEL^+^, indicating that cells with p-RIPK1 (Ser166) were going to die (Fig. 2E). In a recent *in vitro* study, it was reported that Nec-1, but not iNec, specifically inhibited phosphorylated RIPK1 kinase at several residues, including Ser14, Ser15, Ser161 and Ser166 [17]. However, Nec-1 and iNec were also found to inhibit indoleamine 2,3-dixoygenase (IDO), which is implicated in neuroinflammation but not directly associated with RIPK1 activation [29, 30]. To elucidate the mechanism of necroptosis, we further analyzed the effects of Nec-1 and iNec on the activation of RIPK1 and its downstream factors. We found that Nec-1, but not iNec, inhibited activation of RIPK1 and its downstream targets after ischemic stroke. Therefore, we demonstrated that Nec-1 can effectively inhibit RIPK1 phosphorylation at the Ser166 residue since Nec-1, but not iNec, significantly reduces the number of p-RIPK1^+^ neurons (Fig. 4C-D) as well as the expression levels of L-RIPK1 in ischemic injured brains (Fig. 4A-B) although we cannot exclude its inhibitory effects on the other residues. RIPK1 phosphorylation is implicated in caspase-8-mediated apoptosis and RIPK3-MLKL-dependent necroptosis [17, 18, 31]. In this study, we showed that p-RIPK1 (Ser166) induced by ischemia triggered the RIPK3/MLKL-dependent necroptotic pathway. We also demonstrated that ischemia-induced RIPK1-mediated RIPK3/MLKL necroptosis was specific because Nec-1 treatment could significantly inhibit ischemia-induced increase of RIPK3, MLKL and p-MLKL expressions as well as attenuate ischemic brain injury via inhibition of RIPK1 kinase activity (Fig. 4 and Fig. 5A, B-D). Collectively, our results indicate that ischemic-induced necroptotic neuronal death occurs via activation of the RIPK1-mediated RIPK3-MLKL signaling pathway. This study also provides clear-cut biochemical evidence that illustrate the mechanism of the necroptotic process in the ischemic injured brains.

It has been demonstrated that an inflammatory response occurs centrally after ischemic injury [32]. Our results confirm that ischemic injury induced the increase of mature interlukin-1β (IL-1β) in the brain (Fig. 5A, E). Interestingly, Nec-1 treatment could simultaneously inhibit the formation of mature IL-1β in the brain following ischemic stroke. These results suggest that activation of RIPK1-mediated RIPK3/MLKL-dependent necroptosis in ischemic brains could stimulate an inflammatory response, which has a hand in neuronal death. A previous study reported that a reduction of the inflammatory response was seen in transgenic mice with RIPK3 or MLKL kinase genes being deleted [33]. However, when the RIPK1 kinase gene was deleted, there was no change in the inflammatory response [33], but an increase of apoptosis was seen in the liver [34], suggesting that RIPK1 kinase is involved in multiple pathways.

In conclusion, our study was the first to demonstrate that p-RIPK1 (Ser166) increases in the brain after ischemia, which then activated the RIPK3/MLKL-dependent necroptotic signaling pathway to cause neuronal death. Nec-1 treatment was able to protect against ischemic neuronal death by inhibiting RIPK1-mediated RIPK3/MLKL-dependent necroptosis in rat brains following ischemic stroke. Due to the specific activation of p-RIPK1 (Ser166) in cerebral ischemia, we suggest further studies to explore its feasibility as a biomarker for necroptosis and a potential therapeutic target for neuroprotection.

## References

[1] Lipton P (1999). Ischemic cell death in brain neurons. Physiol Rev, 79: 1431-1568. 10508238 10.1152/physrev.1999.79.4.1431

[2] Kalogeris T, Baines CP, Krenz M, Korthuis RJ. 2012 CELL BIOLOGY OF ISCHEMIA/REPERFUSION INJURY. In International Review of Cell and Molecular Biology, Vol 298 Jeon KW, editor. San Diego: Elsevier Academic Press Inc 229-317. 22878108 10.1016/B978-0-12-394309-5.00006-7 PMC3904795

[3] Puyal J, Ginet V, Clarke PGH (2013). Multiple interacting cell death mechanisms in the mediation of excitotoxicity and ischemic brain damage: A challenge for neuroprotection. Prog Neurobiol, 105: 24-48. 23567504 10.1016/j.pneurobio.2013.03.002

[4] Holler N, Zaru R, Micheau O, Thome M, Attinger A, Valitutti S, et al (2000). Fas triggers an alternative, caspase-8-independent cell death pathway using the kinase RIP as effector molecule. Nat Immunol, 1: 489-495. 11101870 10.1038/82732

[5] Degterev A, Huang Z, Boyce M, Li Y, Jagtap P, Mizushima N, et al (2005). Chemical inhibitor of nonapoptotic cell death with therapeutic potential for ischemic brain injury. Nat Chem Biol, 1: 112-119. 16408008 10.1038/nchembio711

[6] Degterev A, Hitomi J, Germscheid M, Ch'en IL, Korkina O, Teng X, et al (2008). Identification of RIP1 kinase as a specific cellular target of necrostatins. Nat Chem Biol, 4: 313-321. 18408713 10.1038/nchembio.83 PMC5434866

[7] Xu XS, Chua KW, Chua CC, Liu CF, Hamdy RC, Chua BHL (2010). Synergistic protective effects of humanin and necrostatin-1 on hypoxia and ischemia/reperfusion injury. Brain Res, 1355: 189-194. 20682300 10.1016/j.brainres.2010.07.080 PMC3412340

[8] Wang YQ, Wang L, Zhang MY, Wang T, Bao HJ, Liu WL, et al (2012). Necrostatin-1 Suppresses Autophagy and Apoptosis in Mice Traumatic Brain Injury Model. Neurochem Res, 37: 1849-1858. 22736198 10.1007/s11064-012-0791-4

[9] You ZR, Savitz SI, Yang JS, Degterev A, Yuan JY, Cuny GD, et al (2008). Necrostatin-1 reduces histopathology and improves functional outcome after controlled cortical impact in mice. J Cereb Blood Flow Metab, 28: 1564-1573. 18493258 10.1038/jcbfm.2008.44 PMC2831087

[10] Chavez-Valdez R, Martin LJ, Flock DL, Northington FJ (2012). Necrostatin-1 attenuates mitochondrial dysfunction in neurons and astrocytes following neonatal hypoxia-ischemia. Neuroscience, 219: 192-203. 22579794 10.1016/j.neuroscience.2012.05.002 PMC4011024

[11] Chavez-Valdez R, Martin LJ, Northington FJ (2012). Programmed Necrosis: A Prominent Mechanism of Cell Death following Neonatal Brain Injury. Neurol Res Int, 2012: 257563. 22666585 10.1155/2012/257563 PMC3362209

[12] Northington FJ, Chavez-Valdez R, Graham EM, Razdan S, Gauda EB, Martin LJ (2011). Necrostatin decreases oxidative damage, inflammation, and injury after neonatal HI. J Cereb Blood Flow Metab, 31: 178-189. 20571523 10.1038/jcbfm.2010.72 PMC3049482

[13] Stanger BZ, Leder P, Lee T-H, Kim E, Seed B (1995). RIP: A novel protein containing a death domain that interacts with Fas/APO-1 (CD95) in yeast and causes cell death. Cell, 81: 513-523. 7538908 10.1016/0092-8674(95)90072-1

[14] Sun X, Yin J, Starovasnik MA, Fairbrother WJ, Dixit VM (2002). Identification of a novel homotypic interaction motif required for the phosphorylation of receptor-interacting protein (RIP) by RIP3. J Biol Chem, 277: 9505-9511. 11734559 10.1074/jbc.M109488200

[15] Cho YS, Challa S, Moquin D, Genga R, Ray TD, Guildford M, et al (2009). Phosphorylation-driven assembly of the RIP1-RIP3 complex regulates programmed necrosis and virus-induced inflammation. Cell, 137: 1112-1123. 19524513 10.1016/j.cell.2009.05.037 PMC2727676

[16] Sun L, Wang H, Wang Z, He S, Chen S, Liao D, et al (2012). Mixed lineage kinase domain-like protein mediates necrosis signaling downstream of RIP3 kinase. Cell, 148: 213-227. 22265413 10.1016/j.cell.2011.11.031

[17] Ofengeim D, Yuan J (2013). Regulation of RIP1 kinase signalling at the crossroads of inflammation and cell death. Nat Rev Mol Cell Biol, 14: 727-736. 24129419 10.1038/nrm3683

[18] Meng H, Liu Z, Li X, Wang H, Jin T, Wu G, et al (2018). Death-domain dimerization-mediated activation of RIPK1 controls necroptosis and RIPK1-dependent apoptosis. Proc Natl Acad Sci U S A, 115(9): E2001-E2009. 29440439 10.1073/pnas.1722013115 PMC5834731

[19] Ofengeim D, Ito Y, Najafov A, Zhang Y, Shan B, DeWitt JP, et al (2015). Activation of Necroptosis in Multiple Sclerosis. Cell Rep. 10(11): 1836-49. 25801023 10.1016/j.celrep.2015.02.051 PMC4494996

[20] Yang ZJ, Bao WL, Qiu MH, Zhang LM, Lu SD, Huang YL, et al (2002). Role of vascular endothelial growth factor in neuronal DNA damage and repair in rat brain following a transient cerebral ischemia. J Neurosci Res, 70: 140-149. 12271463 10.1002/jnr.10380

[21] Longa EZ, Weinstein PR, Carlson S, Cummins R (1989). Reversible middle cerebral artery occlusion without craniectomy in rats. Stroke, 20: 84-91. 2643202 10.1161/01.str.20.1.84

[22] Schallert T, Fleming SM, Leasure JL, Tillerson JL, Bland ST (2000). CNS plasticity and assessment of forelimb sensorimotor outcome in unilateral rat models of stroke, cortical ablation, parkinsonism and spinal cord injury. Neuropharmacology, 39: 777-787. 10699444 10.1016/s0028-3908(00)00005-8

[23] Li J, McQuade T, Siemer AB, Napetschnig J, Moriwaki K, Hsiao YS, et al (2012). The RIP1/RIP3 necrosome forms a functional amyloid signaling complex required for programmed necrosis. Cell, 150: 339-350. 22817896 10.1016/j.cell.2012.06.019 PMC3664196

[24] Chen X, Li W, Ren J, Huang D, He WT, Song Y, et al (2014). Translocation of mixed lineage kinase domain-like protein to plasma membrane leads to necrotic cell death. Cell Res, 24: 105-121. 24366341 10.1038/cr.2013.171 PMC3879712

[25] Cai Z, Jitkaew S, Zhao J, Chiang HC, Choksi S, Liu J, et al (2014). Plasma membrane translocation of trimerized MLKL protein is required for TNF-induced necroptosis. Nat Cell Biol, 16: 55-65. 24316671 10.1038/ncb2883 PMC8369836

[26] Laster SM, Wood JG, Gooding LR (1988). Tumor necrosis factor can induce both apoptic and necrotic forms of cell lysis. J Immunol, 141: 2629-2634. 3171180

[27] Vandenabeele P, Galluzzi L, Vanden Berghe T, Kroemer G (2010). Molecular mechanisms of necroptosis: an ordered cellular explosion. Nat Rev Mol Cell Biol, 11: 700-714. 20823910 10.1038/nrm2970

[28] Xie T, Peng W, Liu Y, Yan C, Maki J, Degterev A, et al (2013). Structural Basis of RIP1 Inhibition by Necrostatins. Structure, 21: 493-499. 23473668 10.1016/j.str.2013.01.016

[29] Degterev A, Maki JL, Yuan J (2013). Activity and specificity of necrostatin-1, small-molecule inhibitor of RIP1 kinase. Cell Death Differ, 20: 366. 23197295 10.1038/cdd.2012.133 PMC3554332

[30] Takahashi N, Duprez L, Grootjans S, Cauwels A, Nerinckx W, DuHadaway JB, et al (2012). Necrostatin-1 analogues: critical issues on the specificity, activity and in vivo use in experimental disease models. Cell Death Dis, 3: e437. 23190609 10.1038/cddis.2012.176 PMC3542611

[31] Dondelinger Y, Jouan-Lanhouet S, Divert T, Theatre E, Bertin J, Gough PJ, et al (2015). NF-kappaB-Independent Role of IKKalpha/IKKbeta in Preventing RIPK1 Kinase-Dependent Apoptotic and Necroptotic Cell Death during TNF Signaling. Mol Cell, 60: 63-76. 26344099 10.1016/j.molcel.2015.07.032

[32] Lambertsen KL, Biber K, Finsen B (2012). Inflammatory cytokines in experimental and human stroke. J Cereb Blood Flow Metab, 32: 1677-1698. 22739623 10.1038/jcbfm.2012.88 PMC3434626

[33] Rickard JA, O'Donnell JA, Evans JM, Lalaoui N, Poh AR, Rogers T, et al (2014). RIPK1 regulates RIPK3-MLKL-driven systemic inflammation and emergency hematopoiesis. Cell, 157: 1175-1188. 24813849 10.1016/j.cell.2014.04.019

[34] Suda J, Dara L (2016). Knockdown of RIPK1 Markedly Exacerbates Murine Immune-Mediated Liver Injury through Massive Apoptosis of Hepatocytes, Independent of Necroptosis and Inhibition of NF-kappaB. J Immunol, 197: 3120-3129. 27605011 10.4049/jimmunol.1600690 PMC5101131

